# Quality of Life and Sleep Among Parents of Children With Autism Spectrum Disorder in Medina

**DOI:** 10.7759/cureus.90153

**Published:** 2025-08-15

**Authors:** Abeer Al-Lihabi, Shouq T Alharbi, Ramzi Alsheraimi, Abdulrahman M Alharbi, Marwa Alofi, Shurouq Azouni, Anfal Albaidani

**Affiliations:** 1 Department of Psychiatry, Taibah University, Medina, SAU; 2 College of Medicine, Taibah University, Medina, SAU; 3 Department of Family Medicine, Health Holdings, Medina, SAU; 4 Department of Pediatric Development and Behavioral Center, Maternity and Children Hospital, Medina, SAU

**Keywords:** autism spectrum disorder (asd), caregiver's mental health, quality of life (qol), quality of sleep, saudi arabia

## Abstract

Introduction: Autism spectrum disorder (ASD) is a common and prevalent neurodevelopmental condition. While the impact of ASD on the child’s well-being is well-documented, there has been limited research on the impact of caring for an autistic child on the sleep patterns and quality of life (QoL) of parents and caregivers. This cross-sectional study aimed to investigate the effects of having an autistic child on the QoL and sleep patterns of parents and caregivers in Medina, Saudi Arabia.

Methods: The study used validated questionnaires to assess sleep quality and QoL, with a sample of 89 participants. The questionnaire was distributed among parents and caregivers of autistic children at local healthcare centers.

Result: The study revealed that the majority of respondents are mothers (70.8%), with children aged 5-7 years being the most common (39.3%), and 67.4% of children are males. Speech problems (47.2%) are the most prevalent issue among autistic children, followed by sleep disorders (20.2%), while a significant portion of parents experience anxiety symptoms (10.1%), depression symptoms (4.5%), and co-occurrence of depression and anxiety symptoms, seen in 5.6% of cases. Parents and caregivers of autistic children experienced moderate levels of sleep disturbance, as indicated by a mean Pittsburgh Sleep Quality Index (PSQI) score of 6.74 ± standard deviation 2.89, with 44.9% of respondents reporting not feeling rested.

Conclusion: The analysis of sleep quality and QoL measures highlighted significant challenges faced by the participants, emphasizing the need for comprehensive support strategies to address the well-being of families affected.

## Introduction

Autism spectrum disorder (ASD) is a frequently occurring, lifelong neurodevelopmental disorder marked by continuous difficulties with social communication, as well as by restricted and repetitive behaviors [1]. The global prevalence of ASD in children between two and four years old is estimated at one in 100 globally and 25 out of 1000 among Saudi children in Riyadh [2,3]. However, the manifestations of ASD are continuous and typically appear in the first three years of life. Clinical manifestations vary in presentation and severity between different patients; however, the majority of autistic children experience some continuous sleep difficulty, such as insomnia and circadian sleep-wake rhythm disorders (CSWRDs) [4]. The prevalence of sleep disorders in autistic children is estimated to be up to 86% compared to 25-40% in neurotypically developing (NT) children. Moreover, half of autistic children have at least one experience of sleep difficulty that appears to be chronic [5,6]. Consequently, parents of autistic children have a higher prevalence of reporting previous sleep difficulties of their children, which is estimated to be around 83% compared to 20-50% of parents of NT children [7]. These lifelong behaviors and sleep problems of autistic children have a negative impact on the quality of sleep (QoS) and psychological well-being of the parents [8]. The number of studies investigating the QoS among parents of autistic children compared to parents of NT children is limited; however, the studies that have been carried out found that parents of autistic children have poorer sleep quality than parents of NT children [9,10]. As was mentioned before, studies show that up to 83% of autistic children consistently experience sleep problems, and this may partially account for the poor sleep habits of parents of autistic children [9]. Inadequate sleep is linked to reduced capacity to control emotions, thoughts, and actions, which can lead to poor decision-making, distractibility, sluggish information processing, impatience, and impulsive behaviors, all of which cause stressful experiences [11]. There is strong evidence supporting the existence of a sleep-stress cycle; in particular, studies of the general population show that experiencing stress negatively impacts sleep quality, which in turn negatively impacts stress [12]. These sleep disturbances and behavioral difficulties not only affect stress levels among parents but also influence their overall quality of life (QoL), a multidimensional concept that reflects individuals’ perceptions of their life in relation to their goals, expectations, standards, and concerns. Therefore, these difficulties can be considered key predictors of parents' overall QoL [13-15].

This study is the first of its kind to have been carried out in Medina on parents and caregivers of autistic children and given the high incidence of autism in the Saudi population and the limited amount of research that has been carried out internationally. The study aims to assess how having an autistic child impacts the QOL and sleep of parents and caregivers.

## Materials and methods

Study design and setting

This is an analytic cross-sectional questionnaire-based study. A self-administered online questionnaire was distributed among parents or caregivers of autistic children at child clinics of King Salman bin Abdulaziz Medical City (KSAMC) in Medina City who were registered in KSAMC’s electronic medical file. Only one response per participant was collected at a single point in time. Although the data collection occurred over a period of several months (from March to December 2023), this was due to logistical reasons related to recruitment and scheduling. No follow-up or repeated measures were conducted; therefore, the study remains cross-sectional in design.

Study population and sampling

The study population consisted of parents or caregivers of autistic children in Medina who were receiving care at centers in the city. The sampling method used was nonprobability convenience sampling. The aim was to obtain the maximum possible number of samples within the available population, based on available data indicating 128 child follow-ups. However, the actual sample size obtained was 89 participants. The inclusion criteria were parents of children diagnosed with ASD and main caregivers of autistic children, including siblings, grandparents, and other relevant caregivers. The inclusion criteria covered all nationalities. The exclusion criteria were parents of autistic children or their caregivers living outside Medina, and autistic children aged over 18 years. The dependent variable was parents and caregivers of autistic children, and the independent variables were QoL and QoS.

Data collection tool

A cross-sectional study was carried out using a self-administered electronic questionnaire (Google Form) distributed to parents of autistic children at the Developmental​ and Behavioral Disorders Center and Child Psychiatry Department at KSAMC and Taiba Medical Specialist Center in Medina City. We used the PubMed database to search for prior related studies using the keywords “quality of life,” “ASD,” and “quality of sleep,” filtering for studies published in the last five years in the Western region of Saudi Arabia. The study used a structured, validated self-administered online questionnaire designed by Google Forms in the Arabic language and distributed among parents and caregivers of autistic children at the Developmental​ and Behavioral Disorders Center and Child Psychiatry Department at KSAMC and Taiba Medical Specialist Center in Medina, using short message service (SMS) messages to collect the information. The questionnaire is divided into three sections based on closed-ended questions. The first section requested informed consent and focused on personal data of the child and caregiver, including sociodemographic data (consanguinity, age, gender, residency and current educational status of the autistic child, marital status of the parents, and income of the family); the child’s medical history (previous diagnoses of psychiatric/developmental disorder, current treatment status), and family history of the child (number of child siblings, previous psychiatric and medical diagnoses of siblings, previous psychiatric diagnoses of the child’s parents). The second section focuses on the Pittsburgh Sleep Quality Index (PSQI), which is a reliable method to assess the quality and quantity of sleep [16,17]. The third section concentrates on the QoL, which was measured using the Arabic version of the World Health Organization’s Quality of Life Brief Version Questionnaire (WHOQOL-BREF) [18,19].

Validity and reliability of the measurement instrument

We used a validated Arabic version of the PSQI, which is a 19-item self-reported questionnaire that asks for information on seven indicators of sleep, including subjective sleep quality, sleep latency, sleep length, habitual sleep efficiency, sleep disruption, use of sleeping pills, and daytime dysfunction, using a scale rated from 0 (no difficulty) to 3 (extreme difficulty, severe difficulty). Also, certain questions have particular arrangements from zero (not during the past month) to three (three or more times a week). A total sleep quality score that ranges from 0 (excellent sleep quality) to 21 (poor sleep quality) is derived by averaging component scores. The overall score that separates “good sleepers” from “poor sleepers” is the total of the scores on the seven subscales. The researchers utilized a cut-off of >5 on the overall PSQI score to differentiate between parents who were “excellent sleepers” and those who were “poor sleepers.” The PSQI has been verified with sufficient psychometric properties in more than 52 distinct languages. The PSQI’s psychometric performance was found to be satisfactory for the Arabic-speaking population in the current study [16,17]. Furthermore, the Arabic version of the WHOQOL-BREF instrument was used to ensure that patients could understand the questions. The WHO Quality of Life Questionnaire has 26 items. Items 1 and 2 can be used to examine a person’s perception of their general health and QoL. Items 3-26 represent four domains, which are subdivided into social relationships (three items), physical health (seven items), psychological health (six items), and environment (eight items). Responses for each item are on a scale of 1 to 5, with 1 indicating the lowest agreement and 5 indicating the highest agreement with that particular item. Items 3, 4, and 26 are negatively formulated and are turned around during analysis [18,19].

Data management and analysis plan

Statistical Analysis

An appropriate statistical analysis was performed using IBM SPSS Statistics for Windows, Version 26 (Released 2018; IBM Corp., Armonk, New York, United States) to calculate QoL and QoS levels. Other statistical analyses were carried out based on the preliminary results.

Data Collection

All participants were asked to fill in the questionnaire.

Method

We conducted tests for data distribution using both the Shapiro-Wilk and Kolmogorov-Smirnov tests. A data distribution was deemed normal if the p-value exceeded 0.05, leading to rejection of the null hypothesis. For quantitative variables, we utilized mean and standard deviation (SD) to provide descriptions, while nonparametric variables were characterized using median and interquartile range (IQR). Categorical variables were summarized using counts and percentages. Pearson's correlation was used to measure the correlation between normally distributed numerical variables. The Mann-Whitney U test and Kruskal-Wallis test were employed to compare the ranks of nonnormally distributed variables.

Ethical considerations

Ethical approval for the study was obtained from the ethical committee of KSAMC (Medina, Saudi Arabia) with IRB Number: IRB23-03 and also from Taibah University, IRB 00010143. Informed consent, in electronic form, was obtained from the participants after explaining the purpose of the research to them. They were informed that participation is voluntary and that all participants will have the option of withdrawing from the study at any time, and they were assured that refusal to participate or withdrawal would not affect the healthcare services provided to them. To ensure the privacy and confidentiality of participants, the data will be accessible only to the primary investigator and co-investigators.

## Results

The “Relation to child” variable shows that mothers represent the majority of the sample, at 70.8%, while fathers constitute 29.2%. Regarding child age, the highest share is between five and seven years (39.3%). Regarding monthly income, nearly a quarter of the participants (25.8%) earn more than 10,000 SAR (approximately 2,667 USD), while a significant portion (22.5%) fall within the 7,500-10,000 SAR range (about 2,000-2,667 USD). In terms of child gender, males constitute 67.4% of the sample, whereas females represent 32.6%. The number of siblings varies, with the largest proportion having one or two siblings (47.2%). Regarding living arrangements, the majority (93.3%) reside with both parents, while only a small percentage live exclusively with either the father (1.1%) or mother (5.6%). Additionally, 11.2% of children do not attend school, and 39.3% follow a daily program (Table 1).

**Table 1:** Demographic characteristics of the studied population

Studied variable (n = 89)		N (%)
Relation to child	Father	26 (29.2%)
	Mother	63 (70.8%)
Child age	Five years or less	18 (20.2%)
	5-7 years	35 (39.3%)
	8-11 years	23 (25.8%)
	12-15 years	6 (6.7%)
	15-18	7 (7.9%)
Monthly income	Less than 2500 SAR (less than 667 USD)	13 (14.6%)
	2500-5000 SAR (approximately 667-1,333 USD)	14 (15.7%)
	5000-7500 SAR (approximately 1,333–2,000 USD)	19 (21.3%)
	7500-10000 SAR (approximately 2,000–2,667 USD)	20 (22.5%)
	More than 10000 SAR (more than 2,667 USD)	23 (25.8%)
Child gender	Female	29 (32.6%)
	Male	60 (67.4%)
Number of siblings	One to two	42 (47.2%)
	Three to four	30 (33.7%)
	Five to six	8 (9.0%)
	More than six	3 (3.4%)
	No siblings	6 (6.7%)
Living	Father	1 (1.1%)
	Mother	5 (5.6%)
	Both parents	83 (93.3%)
Going to school	Do not go to school	10 (11.2%)
	Daily program	35 (39.3%)
	Integration school	13(14.6%)
	Rehabilitation schools	29 (32.6%)
	Public schools	5(6.0%)

**Figure 1:**
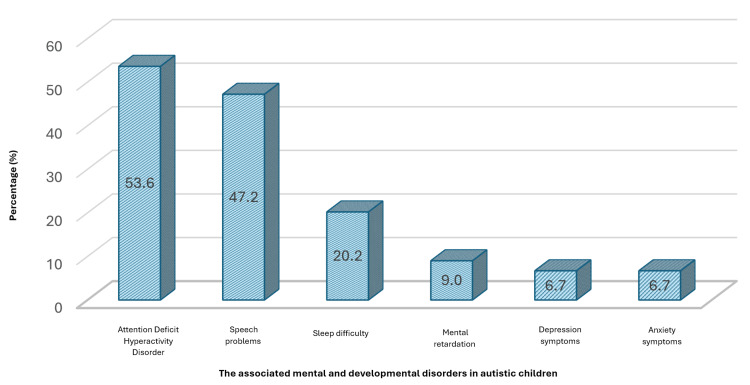
Prevalence of associated mental and developmental disorders in autistic children

Figure 1 shows that speech difficulties emerge as the most prevalent problem, affecting 47.2% of autistic children, indicating a substantial challenge to communication development. Sleep difficulties, identified in 20.2% of cases, suggest a significant impact on sleep patterns and quality of rest. Mental retardation, depression, and anxiety symptoms were also notable, though less prevalent, affecting 9.0%, 6.7%, and 6.7% of autistic children, respectively.

**Figure 2:**
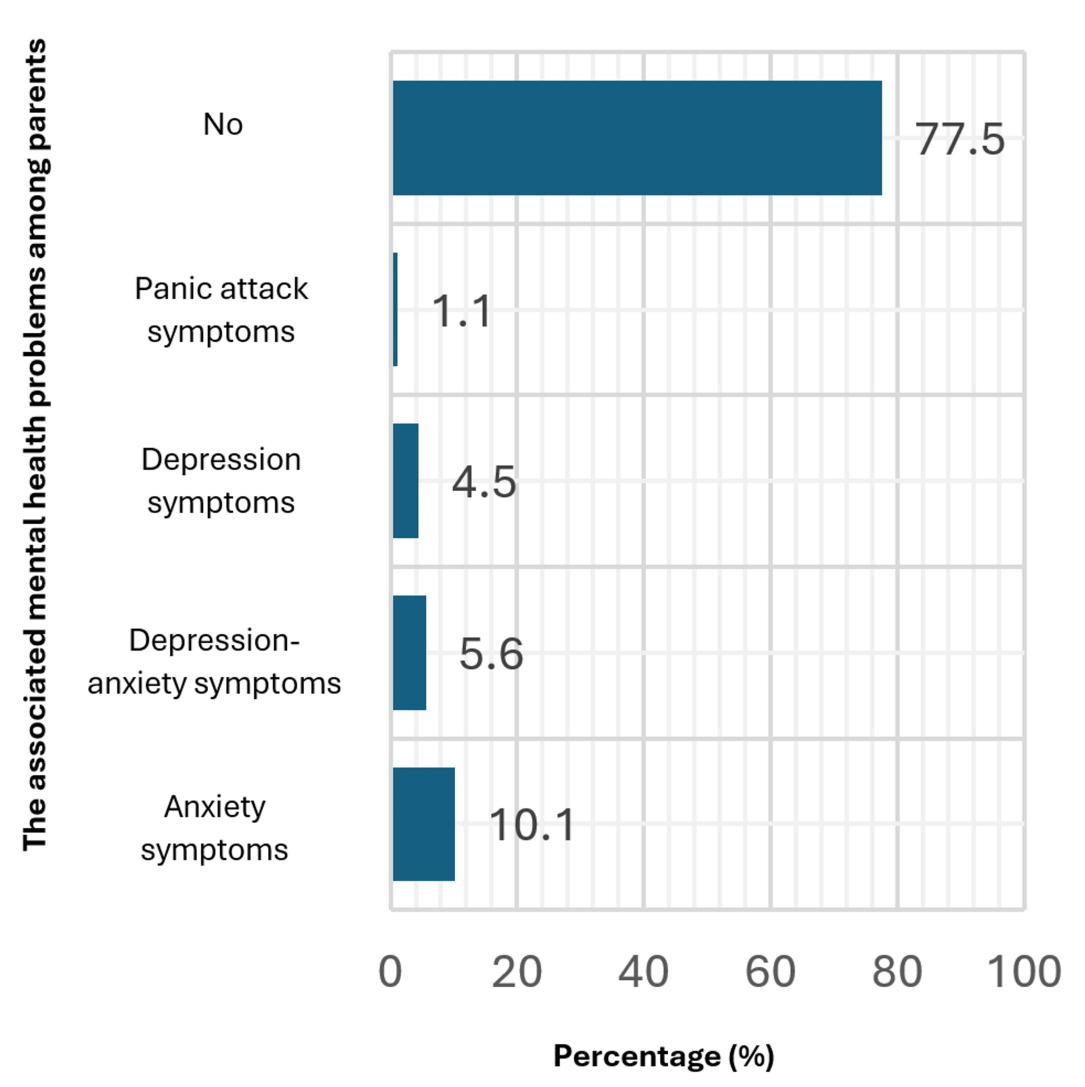
The associated mental health problems among parents

Figure 2 provides insights into the prevalence of mental health problems among parents of autistic children. The data suggests that a majority, comprising 77.5%, report no identified mental health issues. However, anxiety symptoms emerge as the most prevalent condition among those who do report issues, affecting 10.1% of parents. Co-occurrence of depression and anxiety symptoms is observed in 5.6% of cases, while depression symptoms alone are reported by 4.5% of parents. Panic attack symptoms and strokes are relatively less common, each affecting 1.1% of parents.

**Figure 3:**
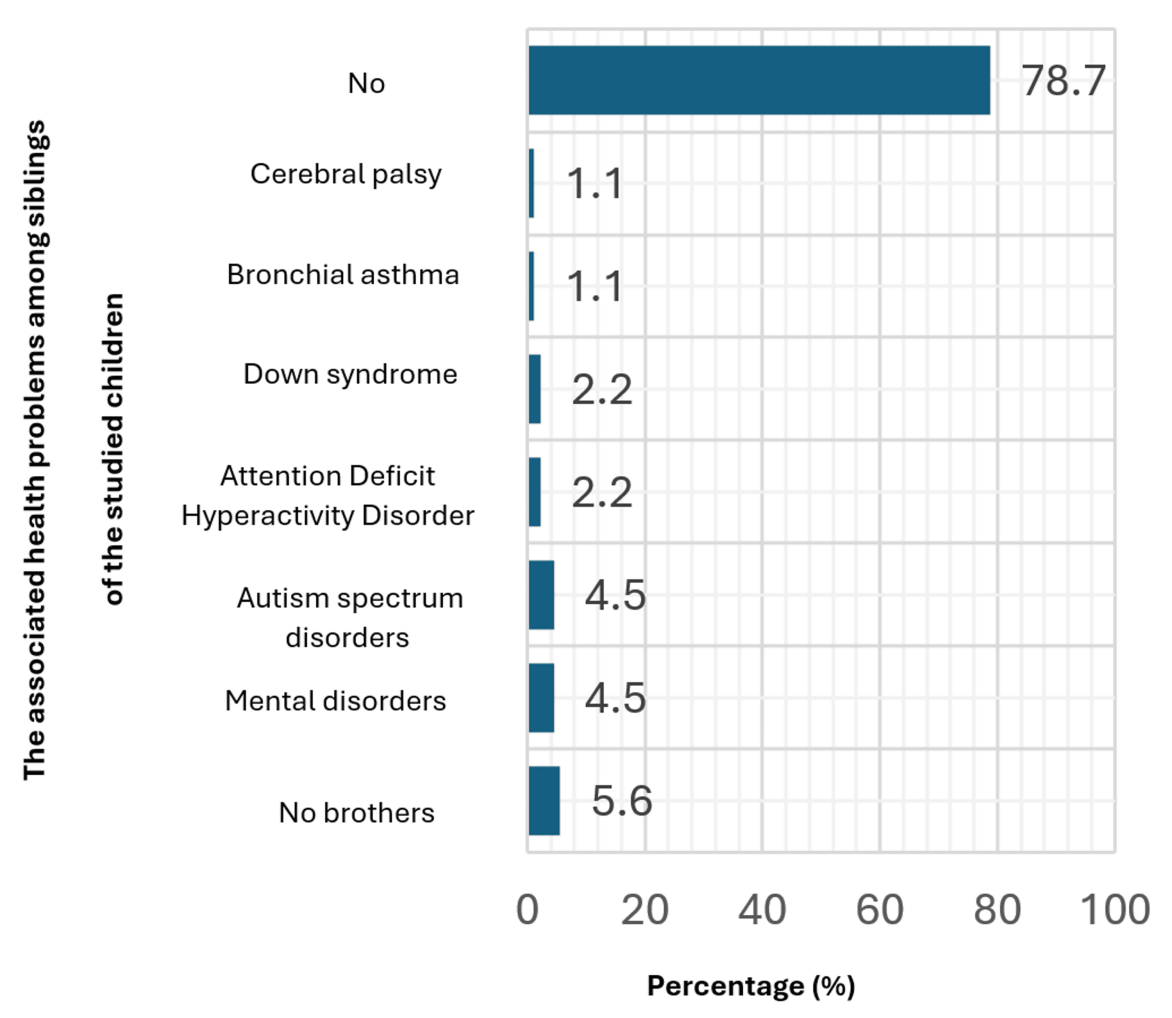
The associated health problems among siblings of the studied children

Figure 3 presents the prevalence of various conditions among siblings within the population. The majority, constituting 78.7%, do not report any identified conditions. However, among those who do report conditions, the absence of brothers is reported by 5.6% of respondents. Mental disorders and autism are each reported by 4.5% of siblings, indicating notable occurrence within this group. Attention deficit hyperactivity disorder (ADHD) and Down syndrome are each observed in 2.2% of cases, while bronchial asthma and cerebral palsy each affect 1.1% of siblings.

**Figure 4:**
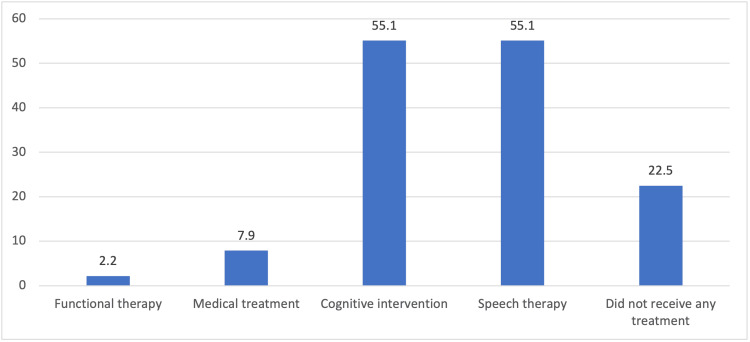
Different treatment modalities that were provided to autistic children

As shown in Figure 4, participants were asked to report the types of treatment their autistic children were receiving. The most commonly utilized interventions included speech therapy and cognitive behavioral intervention, both reported by 55.1% of caregivers. These treatments focus on improving communication skills and behavior management. Less commonly used were medical treatments (7.9%), such as pharmacological support, and functional therapy (2.2%), aimed at enhancing daily living skills. These findings reflect current trends in ASD management.

Most respondents (67.4%) reported having a bed partner, while a smaller percentage indicated having a partner in the same room but not in bed (20.2%), or in another room (5.6%). The frequency of loud snoring varied among respondents, with a significant portion (61.8%) reporting not experiencing loud snoring during the last month. Similarly, a majority (76.4%) reported not experiencing long pauses between breaths while asleep during the last month. Most respondents (67.4%) reported not experiencing leg twitching or jerking while sleeping during the last month, and 60.7% reported not experiencing episodes of disorientation or confusion during sleep during the last month. Finally, a significant portion of respondents (44.9%) reported not feeling rested during the last month (Table 2).

**Table 2:** Sleep habits and disturbances survey results: insights into bed partners, snoring, breathing patterns, and rest quality

Question (N = 89)	Responses	N(%)
Do you have a bed partner or roommate?	Partner in bed	60 (67.4%)
	No partner in bed or never share a room	6 (6.7%)
	Partner in the room, not in bed	18 (20.2%)
	Partner in another room	5 (5.6%)
Loud snoring	Less than once per week	13 (14.6%)
	1-2 times per week	9 (10.1%)
	Three times or more	7 (7.9%)
	Not during the last month	55 (61.8%)
Long pauses between breaths while asleep	Less than once per week	9 (10.1%)
	1-2 times per week	4 (4.5%)
	Three times or more	3 (3.4%)
	Not during the last month	68 (76.4%)
Legs twitching or jerking while you sleep	Less than once per week	9 (10.1%)
	1-2 times per week	10 (11.2%)
	Three times or more	5 (5.6%)
	Not during the last month	60 (67.4%)
Episodes of disorientation or confusion during sleep	Less than once per week	11 (12.4%)
	1-2 times per week	10 (11.2%)
	Three times or more	6 (6.7%)
	Not during the last month	54 (60.7%)
Feeling no rest	Less than once per week	21 (23.6%)
	1-2 times per week	10 (11.2%)
	Three times or more	12 (13.5%)
	Not during the last month	40 (44.9%)

Table 3 shows the mean score of different domains of PSQI. Sleep efficiency shows a mean of 0.45 (SD +/-0.93). Similarly, sleep latency, sleep disturbance, and daytime dysfunction present moderate means of 1.44 (SD +/-0.72), 1.49 (SD +/-0.74), and 0.70 (SD +/-0.82). Sleep duration and use of sleep medication reveal means of 0.97 (SD +/-0.78) and 0.34 (SD +/-0.75). Sleep quality shows a mean of 1.36 (SD +/-1.00). The global PSQI score, with a mean of 6.74 (SD +/-2.89), indicates a moderate level of overall sleep disturbance in the sample.

**Table 3:** Analysis of sleep parameters: mean and variability across domains among parents of autistic children PSQI: Pittsburgh Sleep Quality Index; SD: standard deviation; Q1: first quartile; Q3: third quartile Values are presented as n (%). (a) PSQI score. (b) SD. (c) Q1. (d) Q3

Domains of sleep	Mean	SD^(b)^	Min	Max	Median	Q1^(c)^	Q3^(d)^
Sleep efficiency	0.45	0.93	0.0	3.0	0.0	0.0	0.0
Sleep latency	1.44	0.72	0.0	2.0	2.0	1.0	2.0
Sleep duration	0.97	0.78	0.0	3.0	1.0	0.5	1.0
Sleep disturbance	1.49	0.74	0.0	3.0	1.0	1.0	2.0
Use of sleep medication	0.34	0.75	0.0	3.0	0.0	0.0	0.0
Daytime dysfunction	0.70	0.82	0.0	3.0	1.0	0.0	1.0
Sleep quality	1.36	1.00	0.0	3.0	1.0	1.0	2.0
Global PSQI score^(a)^	6.74	2.89	0.0	16.0	7.0	5.0	9.0

Figure 4 shows common health problems among autistic children: 3.37% had enuresis, 11.24% had dental caries, 12.36% complained of gastrointestinal (GIT) problems, and 16.85% had sensory problems.

**Figure 5:**
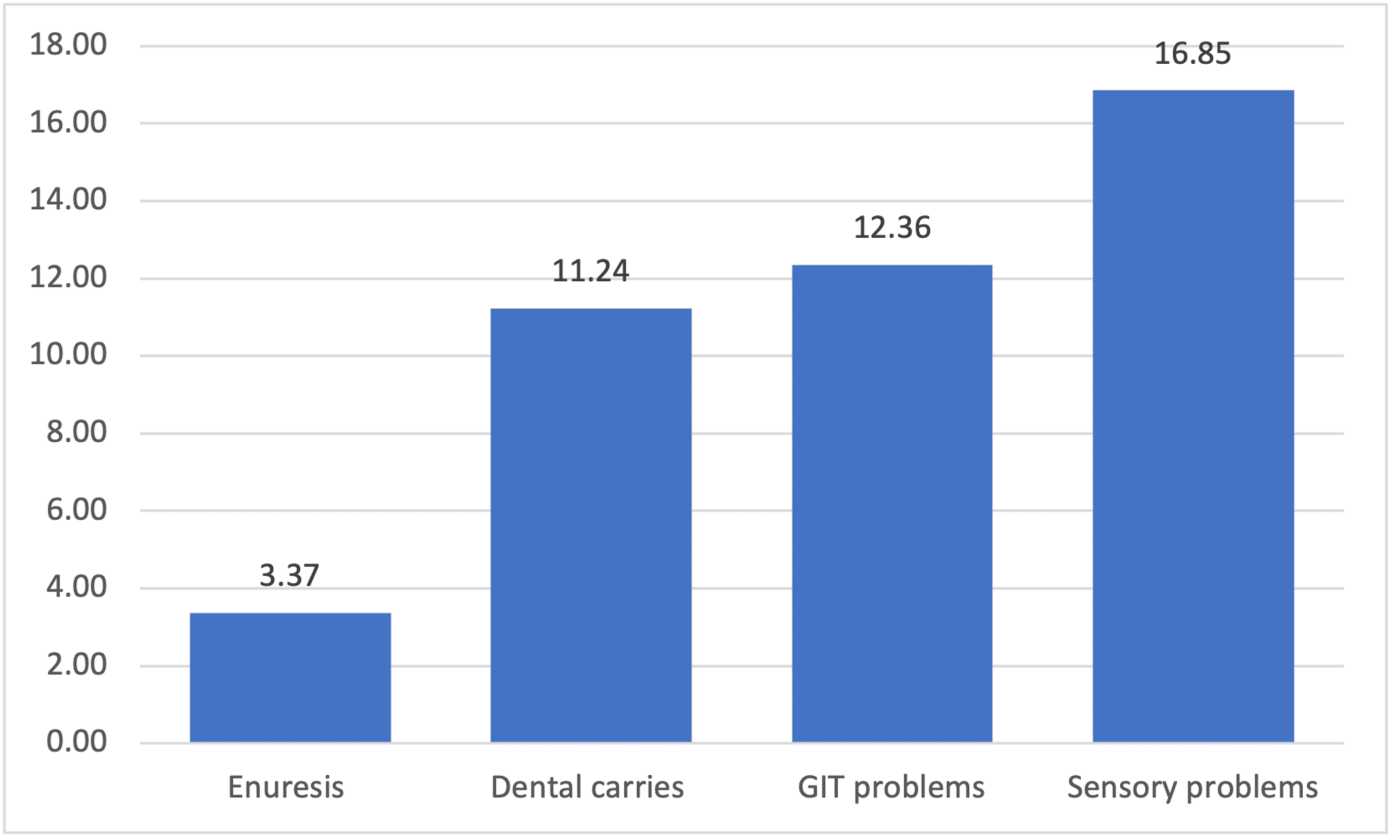
Health problems among autistic children

For QoL assessment (Table 4), participants report a mean score of 3.09 (SD +/-0.96), indicating a moderate level of perceived QoL. General health scores are slightly higher with a mean of 3.28 (SD +/-1.11), suggesting a relatively positive perception of overall health among participants. In specific domains, physical well-being demonstrates a mean of 56.94 (SD +/-20.98), indicating a moderately positive assessment, while psychological and social relations exhibit means of 51.50 (SD +/-18.15) and 51.12 (SD +/-28.13), respectively, indicating moderate levels of psychological and social well-being. The environment domain, with a mean of 48.10 (SD +/-18.70), indicates a moderate satisfaction level with environmental factors.

**Table 4:** Quality of life assessment: domain-specific analysis among parents of autistic children SD: standard deviation; (a) QoL: quality of life Values are presented as n (%)

Domains of QoL^(a)^	Mean	SD	Min	Max	Median	Q1	Q3
General QoL	3.09	0.96	1.0	4.0	3.0	3.0	4.0
General health	3.28	1.11	1.0	5.0	4.0	2.0	4.0
Physical	56.94	20.98	10.7	96.4	60.7	42.9	71.4
Psychological	51.50	18.15	4.2	83.3	54.2	41.7	66.7
Social relations	51.12	28.13	0.0	100.0	58.3	25.0	75.0
Environment	48.10	18.70	0.0	87.5	50.0	37.5	62.5

Regarding sleep quality across age, sex of the child, and relation of the caregiver, whether father or mother, male or female, the data showed that there were no statistically significant differences with p-values > 0.05.

Regarding the QoL across age, sex of the child, and the relationship of the caregiver, whether father or mother, male or female. The study found that there were no statistically significant differences, with all p-values > 0.05.

## Discussion

The current study sheds light on the effect of multiple life aspects of autistic children on their caregivers’ QoL and QoS. Previous research showed that in most cases, the primary caregivers of autistic children were mothers, reflecting demographic characteristics of the population [13]. This finding is consistent with our findings that most caregivers (70.8%) were mothers. The gender gap in caregiving obligations may be ascribed to cultural norms and expectations, as well as the greater frequency of ASD among male children (67.4%) in the sample, which aligns with the well-established gender disparity in ASD diagnosis [20].

According to the study, speech issues were the most common additional condition observed in autistic children, affecting 47.2% of them. Sleep abnormalities were the second most prevalent, affecting 20.2% of the children. The results of this study align with previous research that has emphasized the elevated prevalence of communication challenges and sleep disruptions in autistic children [4,6]. Deficiencies in communication can significantly impact a child’s ability to explain their needs, interact with others, and engage in daily activities, which can lead to increased stress and frustration for both the child and their caregivers [21]. Autistic children may exhibit worsening of their behavioral and emotional difficulties if they experience sleep disturbances such as sleeplessness and issues with the body’s natural sleep-wake cycle [4]. Furthermore, according to Herrmann, these disturbances can also result in sleep deprivation and fatigue among the parents of autistic children [5].

Approximately 77.5% of parents indicated that they did not have any discernible mental health issues. The most prevalent mental health difficulties reported in the study population were anxiety symptoms, which affected 10.1% of individuals. Additionally, 5.6% of the sample reported the co-occurrence of depression and anxiety symptoms, while 4.5% reported depressive symptoms alone.

The results emphasize the considerable emotional burden of taking care of an autistic child, as well as the heightened likelihood of parents and caregivers developing mental health difficulties. Persistent stress, which can emerge as anxiety and depression symptoms, can be caused by several factors, including the responsibilities of maintaining continual monitoring, addressing problematic behaviors, and navigating sophisticated healthcare and educational systems [18,22]. Furthermore, social disapproval and isolation that are associated with autistic children may exacerbate the feelings of isolation and psychological suffering that their parents experience [12].

The analysis of sleep parameters revealed that parents had moderate levels of sleep disturbance, as evidenced by a mean PSQI score of 6.74 (SD +/- 2.89). This finding corroborates previous research that discovered lower sleep quality in parents of autistic children compared to parents of typically developing children [9-10]. Autistic children often have sleep disturbances, including trouble falling asleep, frequent awakenings during the night, and waking up early in the morning. According to a previous study, these interruptions might interfere with parents’ sleep habits and lead to sleep deprivation over a more extended period [6]. Inadequate sleep can substantially influence parents’ physical health, cognitive ability, and emotional control, reducing their capacity to bear parenting obligations and increasing the amount of stress that they experience [12,23].

The QoL survey indicated moderate levels of well-being in several aspects. Among these, physical well-being had the highest rating, 56.94 (SD +/- 20.98), while the environment had the lowest score, 48.10 (SD +/- 18.70). The results suggest that even if parents have good physical health, taking care of an autistic child may negatively affect their emotional, social, and environmental well-being [14,24-25]. The low scores in the environment domain may indicate the challenges parents have in accessing suitable resources, accommodation, and support in their communities, as well as the financial strain of caring for an autistic child [13]. According to Mihaila and Hartley, average scores in the domains of psychological and social connection can be taken as an indicator of the emotional and social implications of ASD for parents. These effects include feelings of isolation, stigma, and strain in relationships [8].

The study's findings revealed no statistically significant changes in QoL or sleep quality associated with demographic data, including age, child's gender, or caregiver connection (father or mother). This might suggest that different subgroups of parents and caregivers experience the same difficulties and results when they are caring for an autistic child. Nevertheless, it is essential to recognize that individual experiences might differ significantly. According to the qualitative interviews done in this Riyadh study, the obstacles faced by these caregivers of autistic children ranged from interpersonal to economic, social, institutional, and psychological [25].

Limitations and recommendations

This study has certain limitations, primarily due to the sample size, which was informed by the available population data from the developmental disorder center at KSAMC, where 128 children receive follow-up care. Conducting research within this specific population posed challenges, including limited accessibility to participants and time constraints, especially given the scarcity of developmental centers in Medina. We were ultimately able to include 89 participants, which, while modest, provides preliminary insights into the QoL and sleep patterns of parents and caregivers of autistic children in Medina. Additionally, this study relied on self-reported questionnaires, which, despite being validated and widely used, may be subject to recall bias and subjectivity. The ASD diagnoses were made by qualified clinicians based on the Diagnostic and Statistical Manual of Mental Disorders, 5th Edition (DSM-5 criteria); however, gold-standard diagnostic tools such as the Autism Diagnostic Observation Schedule (ADOS) and Autism Diagnostic Interview-Revised (ADI-R) were not consistently used, and ASD severity was not assessed. Objective sleep assessments, such as actigraphy or polysomnography, were also not incorporated. Future studies should consider longitudinal designs including standardized diagnostic instruments, ASD severity stratification, and objective sleep measures to provide a more comprehensive understanding of the long-term impact of ASD on caregiver well-being.

## Conclusions

This study examines the impact of having an autistic child on the QoL and sleep quality of parents and caregivers in the city of Medina in Saudi Arabia. The results suggest that parents, especially mothers, of autistic children have substantial challenges, including low sleep quality and psychological strain. The issues are exacerbated by the behavioral and sleep difficulties often associated with ASD, leading to a greater prevalence of sleep disorders among parents of autistic children compared to parents of NT children. The study highlights the need for customized treatments and support systems to reduce stress and improve the QoS for parents of autistic children. This is crucial not just for the parents’ welfare but also for the appropriate nurturing and supervision of their children.

## References

[1] American Psychiatric Association (2022). Autism spectrum disorder. Diagnostic and Statistical Manual of Mental Disorders, Fifth Edition.

[2] Zeidan J, Fombonne E, Scorah J (2022). Global prevalence of autism: a systematic review update. Autism Res.

[3] AlBatti TH, Alsaghan LB, Alsharif MF (2022). Prevalence of autism spectrum disorder among Saudi children between 2 and 4 years old in Riyadh. Asian J Psychiatr.

[4] Schreck KA, Richdale AL (2020). Sleep problems, behavior, and psychopathology in autism: inter-relationships across the lifespan. Curr Opin Psychol.

[5] Herrmann S (2016). Counting sheep: sleep disorders in children with autism spectrum disorders. J Pediatr Health Care.

[6] Singh K, Zimmerman AW (2015). Sleep in autism spectrum disorder and attention deficit hyperactivity disorder. Semin Pediatr Neurol.

[7] Elrod MG, Nylund CM, Susi AL, Gorman GH, Hisle-Gorman E, Rogers DJ, Erdie-Lalena C (2016). Prevalence of diagnosed sleep disorders and related diagnostic and surgical procedures in children with autism spectrum disorders. J Dev Behav Pediatr.

[8] Mihaila I, Hartley SL (2018). Parental sleep quality and behavior problems of children with autism. Autism.

[9] Meltzer LJ (2008). Brief report: sleep in parents of children with autism spectrum disorders. J Pediatr Psychol.

[10] Lopez-Wagner MC, Hoffman CD, Sweeney DP, Hodge D, Gilliam JE (2008). Sleep problems of parents of typically developing children and parents of children with autism. J Genet Psychol.

[11] Vandekerckhove M, Cluydts R (2010). The emotional brain and sleep: an intimate relationship. Sleep Med Rev.

[12] Doane LD, Thurston EC (2014). Associations among sleep, daily experiences, and loneliness in adolescence: evidence of moderating and bidirectional pathways. J Adolesc.

[13] Hartley SL, Seltzer MM, Head L, Abbeduto L (2012). Psychological well-being in fathers of adolescents and young adults with Down syndrome, Fragile X syndrome, and autism. Fam Relat.

[14] Lecavalier L, Leone S, Wiltz J (2006). The impact of behaviour problems on caregiver stress in young people with autism spectrum disorders. J Intellect Disabil Res.

[15] Karimi M, Brazier J (2016). Health, health-related quality of life, and quality of life: what is the difference?. Pharmacoeconomics.

[16] Suleiman KH, Yates BC, Berger AM, Pozehl B, Meza J (2010). Translating the Pittsburgh Sleep Quality Index into Arabic. West J Nurs Res.

[17] Alqarni RA, Almutairi NS, Albalawi AS, Alsulami MB, Alhashrani MA, Bin Abdulrahman KA (2025). Test-retest reliability of the Arabic version of the Pittsburgh Sleep Quality Index. Medicine (Baltimore).

[18] (2025). WHOQOL-BREF. https://www.who.int/tools/whoqol/whoqol-bref/docs/default-source/publishing-policies/whoqol-bref/arabic-whoqol-bref.

[19] Ohaeri JU, Awadalla AW (2009). The reliability and validity of the short version of the WHO Quality of Life Instrument in an Arab general population. Ann Saudi Med.

[20] Loomes R, Hull L, Mandy WP (2017). What is the male-to-female ratio in autism spectrum disorder? A systematic review and meta-analysis. J Am Acad Child Adolesc Psychiatry.

[21] Sharma N, Chakrabarti S, Grover S (2016). Gender differences in caregiving among family-caregivers of people with mental illnesses. World J Psychiatry.

[22] Tathgur MK, Kang HK (2021). Challenges of the caregivers in managing a child with autism spectrum disorder-a qualitative analysis. Indian J Psychol Med.

[23] Schwichtenberg AJ, Janis A, Lindsay A (2022). Sleep in children with autism spectrum disorder: a narrative review and systematic update. Curr Sleep Med Rep.

[24] Lone MA, Almakeynah MA, Alsahaf HA, Alalawi MH, Aldhneen BAA, Aldabbab MA, Alghanim ME (2022). Quality of life among parents of children with autism spectrum disorder in riyadh. IJMDC.

[25] Alshaigi K, Albraheem R, Alsaleem K, Zakaria M, Jobeir A, Aldhalaan H (2020). Stigmatization among parents of autism spectrum disorder children in Riyadh, Saudi Arabia. Int J Pediatr Adolesc Med.

